# Two-dimensional semiconductors integrated with hybrid dielectrics for post-Moore electronics

**DOI:** 10.1093/nsr/nwad266

**Published:** 2023-10-12

**Authors:** Wei Zhai, Yao Yao, Zijian Li, Li Zhai, Hua Zhang

**Affiliations:** Department of Chemistry, City University of Hong Kong, China; Department of Chemistry, City University of Hong Kong, China; Department of Chemistry, City University of Hong Kong, China; Department of Chemistry, City University of Hong Kong, China; Hong Kong Branch of National Precious Metals Material Engineering Research Center (NPMM), City University of Hong Kong, China; Department of Chemistry, City University of Hong Kong, China; Hong Kong Branch of National Precious Metals Material Engineering Research Center (NPMM), City University of Hong Kong, China; Shenzhen Research Institute, City University of Hong Kong, China

As the basic building blocks of modern electronics, field-effect transistors (FETs) use gate electrodes to control the conductivity of channel materials through the electrical polarization of dielectrics [1]. The performance of FETs relies not only on the channel materials and their contacts with the electrodes, but also on the dielectrics and their interfaces with the channel materials [1,2]. According to Moore's law, FETs composed of conventional bulk semiconductors are approaching their physical limits and new semiconducting materials are thereby highly demanded. Recently, 2D semiconductors have shown great potential as promising channel materials to replace conventional bulk semiconductors for next-generation FETs due to their atomically thin thickness and dangling-bond-free characteristics [2]. To date, great efforts have been made in the design and preparation of 2D semiconductor-based FETs with desirable performance. However, it remains challenging to fabricate dielectrics with high-quality interfaces in 2D semiconductor-based FETs using processes that are compatible with the complementary metal-oxide-semiconductor (CMOS) technology [1].

**Figure 1.**
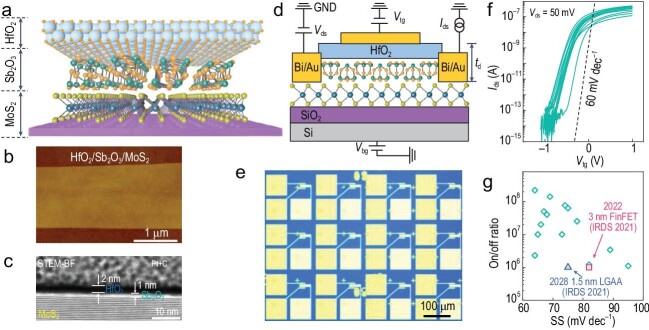
FETs fabricated with MoS_2_ and hybrid HfO_2_/Sb_2_O_3_ dielectric layer. (a) Schematic diagram of the fabricated HfO_2_/Sb_2_O_3_ hybrid dielectric layer on MoS_2_. (b) Atomic force microscope image of HfO_2_/Sb_2_O_3_ on MoS_2_. (c) Bright-field cross-sectional scanning transmission electron microscopy image of HfO_2_/Sb_2_O_3_ on MoS_2_. (d) Schematic illustration of the FET structure with the hybrid HfO_2_/Sb_2_O_3_ layer as the gate dielectric. (e) Optical image of FET arrays fabricated with a MoS_2_ monolayer and a HfO_2_/Sb_2_O_3_ hybrid dielectric layer. (f) Transfer characteristic curves of FET arrays fabricated with a MoS_2_ monolayer and a HfO_2_/Sb_2_O_3_ hybrid dielectric layer. (g) The statistics of SS values and on/off ratios of FET arrays. Reprinted with permission from [3].

Recently, the Zhai research group reported a versatile method to prepare high-quality dielectrics on 2D semiconductors with sub-1-nm equivalent oxide thickness (EOT) [3]. Specifically, a thermally evaporated inorganic molecular crystal, Sb_2_O_3_, was innovatively introduced as the buffer layer between the 2D semiconducting MoS_2_ and the ultra-thin atomic-layer-deposited high-*κ* HfO_2_ layer (Fig. 1a–d). Sb_2_O_3_ can form a high-quality interface with MoS_2_ due to its cage-like molecular structure without dangling bonds [4]. Moreover, the hydrophilicity of Sb_2_O_3_ facilitates the precursor adsorption during atomic layer deposition of HfO_2_, resulting in the uniform deposition of the high-*κ* HfO_2_ dielectric layer. The hybrid dielectric layer composed of Sb_2_O_3_ and high-*κ* HfO_2_ has a high-quality interface with MoS_2_ and an excellent dielectric property with a high gate capacitance of 3.2–3.5 μF/cm^2^. The performance of FET arrays (Fig. 1e) fabricated with the MoS_2_ monolayer and the hybrid HfO_2_/Sb_2_O_3_ dielectric layer proves that their approach has good reproducibility and device uniformity. The fabricated top-gated FETs exhibit an average subthreshold swing (SS) value of 73 mV/dec and an on/off ratio of 5 × 10^7^ (Fig. 1f and g), which are comparable to the state-of-the-art Si-based transistors. Especially, this approach is compatible with CMOS technology, holding great promise for industrial applications in post-Moore electronics.

The research work [3] demonstrates the key role of the dielectric design in boosting the performance of 2D semiconductor-based FETs for post-Moore electronics. More functional 2D semiconductor-based devices fabricated with various hybrid dielectrics are anticipated. In addition to the dielectric layer, the contact between the channel material and the electrode also plays a critical role in determining the performance of FETs. Recently, phase engineering of nanomaterials (PEN) [5] has been used as an effective strategy to reduce contact resistances and improve the performance of FETs [6]. We believe that the combination of dielectric engineering and PEN paves the way toward practical applications of 2D semiconductor-based FETs in post-Moore electronics.
